# Effects of Adding Eubiotic Lignocellulose on the Growth Performance, Laying Performance, Gut Microbiota, and Short-Chain Fatty Acids of Two Breeds of Hens

**DOI:** 10.3389/fvets.2021.668003

**Published:** 2021-09-13

**Authors:** Baosheng Sun, Linyue Hou, Yu Yang

**Affiliations:** ^1^Laboratory of Poultry Production, College of Animal Science, Shanxi Agricultural University, Jinzhong, China; ^2^Department of Modern Agriculture, Zunyi Vocational and Technical College, Zunyi, China

**Keywords:** eubiotic lignocellulose, hens, gut microbiota, short-chain fatty, laying performance, dietary fiber, Bian chicken, ISA brown chicken

## Abstract

Eubiotic lignocellulose is a new and useful dietary fiber source for chickens. However, few studies have been undertaken on the impacts of its use as a supplement in different chicken breeds. In this experiment, 108 Chinese native breed Bian hens (BH) and 108 commercial breed ISA Brown hens (IBH) were chosen. They were randomly divided into three groups, and 0, 2, or 4% eubiotic lignocellulose was added to their feed during the growing periods (9–20 weeks), respectively. We aimed to observe the impacts of adding eubiotic lignocellulose on the growth and laying performance, gut microbiota, and short-chain fatty acid (SCFA) of two breeds of hens. In this study, the addition of eubiotic lignocellulose had no significant effect on the growth performance and gut microbial diversity in the two breeds of chickens (*P* > 0.05). Compared with the control group, adding 4% eubiotic lignocellulose significantly increased the cecum weight, laying performance (*P* < 0.05), but had no significant effect on the SCFA of BH (*P* > 0.05); however, adding 4% significantly inhibited the intestinal development, laying performance, butyrate concentration, and SCFA content of IBH (*P* < 0.05). Moreover, the relative abundances of the fiber-degrading bacteria *Alloprevotella* and butyrate-producing bacteria *Fusobacterium* in the 4% group of BH were significantly higher than those in the 4% group of IBH (*P* < 0.05), resulting in the concentration of butyrate was significantly higher than those in it (*P* < 0.05). Combining these results suggests that the tolerance of BH to a high level of eubiotic lignocellulose is greater than that of IBH and adding 2-4% eubiotic lignocellulose is appropriate for BH, while 0–2% eubiotic lignocellulose is appropriate for IBH.

## Introduction

Dietary fiber is important for the growth and production performance of chickens. Studies have shown that adding 1% insoluble fiber to the diet of 8 weeks old Hy-Line brown hens significantly increased the feed utilization rate (1). Added chito-oligosaccharide to layer diets improved the digestibility of dry matter and crude protein (2). Adding mannan-oligosaccharides at the levels of 1 and 1.5 g/kg of diet improved the laying performance and feed efficiency of old hens (3). Dietary fiber can also affect the development of chicken's gastrointestinal tract. Fiber intake can stimulate gizzard function and increase the relative length and weight of chicken cecum (4). However, the tolerance of different chicken breeds to dietary fiber is usually different. It reported that high fiber level significantly reduced the daily gain of broilers, but had no effect on the daily gain of layers (5). In addition, dietary fiber is a source of carbon source and energy for gut microorganisms. The consumption of a high-fiber diet helps to increase the richness and diversity of the gut microbiota (6). Adding chicory extract to the diet of laying hens significantly reduced the number of cecal harmful bacteria (7). The gut microbiota in different chicken breeds was usually different. Distinct differences in gut microbiota between Indian native chickens and commercial chickens have been observed (8).

Among various dietary fiber supplementation, eubiotic lignocellulose has been proven to be a new and effective dietary fiber source for chickens. It is made from special pure wood, and it is a synergistic combination of soluble fiber and insoluble fiber. Its fiber content is as high as 85%, so no great adjustment is required if we want to increase the dietary fiber level of the feed (9). Generally, adding 1.0–1.5% can positively affect the digestion process of poultry. Research has shown that adding 1–1.5% eubiotic lignocellulose to broilers increases their feed intake by 6.7–9.4% (10). Adding 1% eubiotic lignocellulose from the 50th week of life was found to increase the laying production of hens by 1.7% and reduce the feed waste rate by 20% (9).

However, monogastric animals, such as chickens, humans (11), and even pandas (12), lack endogenous fiber-degrading enzymes and must rely on gut microorganisms to degrade dietary fiber, including eubiotic lignocellulose. The members of the Bacteroidetes phylum are “generalists” that degrade dietary fiber polysaccharides. It can utilize a wide range of dietary polysaccharides from plant sources *via* unique polysaccharide utilization locus (PUL) (13, 14). *Bacteroides* and *Prevotella* are excellent fiber-degrading bacterial genera belonging to the Bacteroidetes phylum. *Bacteroides thetaiotamicron* is one of the best fiber-degrading species, and it can degrade the most complex glycan, rhamnogalacturonan-II (RG-II) (15). In contrast, the members of the Firmicutes phylum are regarded as “specialists” for fiber-degradation. The excellent fiber-degrading genera in Firmicutes include *Ruminococcus, Fibrobacter* (16), *Clostridium* and *Roseburia* (17) etc. *Ruminococcus* can degrade dietary fiber into monosaccharides through cellulase or the cellulosome mechanism (18). Some monosaccharides can be used as a carbon source and as energy for gut microbial growth, thus increasing the microbial diversity (7). The leftover monosaccharides enter the cytoplasm and are then fermented to produce short-chain fatty acids (SCFAs) by SCFA-producing bacteria, mainly including acetate, propionate, and butyrate, which account for 90–95% of SCFAs. Cecum is the main site for microbial fiber-degradation and fermentation in chickens. Pathways for the biosynthesis of SCFAs from dietary fiber fermentation differ (19). *Bifidobacterium* produces acetate using the bifid-shunt way (20). Acetate is the main way for the body to obtain energy from dietary fiber. *Propionibacterium* can produce propionate by a succinate-propionate pathway (21) and it synthesize glycogen in the liver. *Faecalibacterium* can ferment glucose into butyrate (22). Butyrate provides ~70% energy for normal colonic epithelial cells (23).

Therefore, we speculated that the previous observed effect of eubiotic lignocellulose on the growth and laying performance of chickens may be closely related to gut microorganisms and their metabolites—SCFAs—but there is limited related research available. In addition, there have been few studies on the impact of adding eubiotic lignocellulosese to different chicken breeds. Given this, a total of 108 Chinese native breed Bian hens (BH) and 108 commercial breed ISA Brown hens (IBH) were chosen for study because the tolerance of Chinese native breed to dietary fiber is usually better than that of commercial breed. They were randomly divided into three groups which were given feed containing three levels of added eubiotic lignocellulose (0, 2, and 4%) for 9–20 weeks. Our aims were to evaluate the impacts of adding eubiotic lignocellulose on the growth performance, laying performance, gut development, gut microbiota, and SCFAs of two breeds of chickens; determine an appropriate additive amount of eubiotic lignocellulose; and provide a theoretical basis for the application of this new feed additives.

## Materials and Methods

### Experiment Design and Animal Management

This experiment was approved by the Shanxi Agricultural University Animal Experiment Ethics Committee (license number: SXAU-EAW-2017-002Chi.001). In total, 108 Chinese native breed BH and 108 commercial breed IBH were chosen. Each breed was randomly divided into three groups. Each group owned six cages with six chickens per cage. One cage was a replicate. These three groups were fed 0, 2, or 4% eubiotic lignocellulose OptiCell (OC) to the basic diets (Table 1) during growing periods (9-20 weeks). Groups one and two were named the OC-low (OL) group and OC-high (OH) group, respectively. The control group was referred to as the OC-free (OF) group. Samples were harvested to measure the gut microbiota, the concentration of SCFAs, and other parameters at the end of the 20-week study period.

**Table 1:** Ingredients and nutrient levels of diets used during weeks 9–20.

**Item**	**Ingredients (%)**
Corn	60.49
Soybean meal	10
Bran	8.5
Spray corn husk	6.5
Distillers dried grains with solubles (DDGS)	5.75
White stone power	2.1
Zeolite	2
Monosodium glutamate	2
CaHPO_4_	0.7
Soybean oil	0.5
Multivitamin^a^	0.35
NaCI	0.28
Minerals^b^	0.5
Hemoglobin powder	0.1
Lys	0.08
Met	0.06
Choline chloride	0.05
Thr	0.04
Total	100
Nutrient levels^c^	
ME	11.40 (MJ/kg)
Crude protein	15.3
Crude ash	5.67
Crude fat	3.99
Crude fiber	3.95
Ca	0.99
Total P	0.5
NaCI	0.37

a *Per kilogram of premix contained vitamin A 13,000-19,000 IU; vitamin B_1_ ≥ 24 mg; vitamin B_2_ ≥ 100 mg; vitamin B_5_ ≥ 200 mg; vitamin B_6_ ≥ 60 mg; vitamin B_12_ ≥ 200 mg; vitamin D_3_ 30,000-90,000 IU; vitamin E ≥ 350 IU; vitamin K_3_ ≥ 60 mg; nicotinamide ≥ 550 mg; folic acid ≥ 12 mg; biotin ≥ 2 mg*.

b *Per kilogram of premix contained Fe 1,300-7,400 mg; Cu 120-650 mg; Mn 1,450-2,900 mg; Zn 1,250-2,900 mg; I 7−95 mg; Se 6-9.5%; Ca 12-25%; P (adding phytase) ≥ 2.0%; NaCI 4-10%; methionine ≥ 1.8%; moisture ≤ 10%*.

c *The composition was calculated but not measured for the diet use*.

The bought eubiotic lignocellulose (Beijing e-feed and e-vet cooperation, Beijing, China) in this study was developed by Agromed Ltd. (Austria). It was made from special fresh timber. It contains total dietary fiber (TDF) 88%, crude fiber 59%, soluble TDF 1.3%, NDF 78%, ADF 64%, lignin 25-30%, energy ~0%, crude protein 0.9%, crude fat 0.8%, moisture 8%, crude ash 1.0%, minerals and trace elements 1.3%.

Chickens were given free access to water and diet. The management of the temperature, light, and humidity was carried out according to the breeding manual of IBH. No immunization schedule was performed to avoid impacts on the gut microbiota of chickens.

### Sampling

The body weight and feed intake of each group of chickens were recorded. The age of laying the first egg in each group was recorded, and all eggs laid on each day in every replicate were collected for seven weeks. Chickens with similar weights from each replicate per group (*n* = 6) were chosen at 20 weeks old. They were slaughtered humanely using the oral bloodletting slaughtering method and then the length and weight of their gastrointestinal tracts were measured. The left cecum chyme of each chicken was squeezed into a multiple cryogenic tube that had been set to zero, and weighed. The cryogenic tubes were quickly placed into a liquid nitrogen tank and stored at −80°C for DNA extraction (24) and the 16S rRNA gene sequence of the gut microbiota and the determination of SCFAs.

### Measurements

#### Growth Performance

The average daily feed intake (ADFI) per chicken per group was computed. ADFI = total feed consumption per week ÷ seven days ÷ numbers of chickens. The average daily gain (ADG) per chicken per group was calculated as follows: ADG = (weights of chickens this week—weights of chickens last week) ÷ seven days ÷ number of chickens.

#### Laying Performance

The age of at which the first egg was laid and the number of eggs laid per replicate were recorded. The average weekly egg weight per group was calculated. Data pertaining to the weights of softshell eggs, and eggs that were too large or too small, were removed. In addition, the average daily laying rate per group was calculated. Laying rate (%) = the total number of eggs per week per group ÷ seven days ÷ number of chickens per group.

#### Development of the Gastrointestinal Tract

The weight or length of the crop, gizzard, small intestine, and cecum were measured. The crop rate and gizzard rate were calculated. Crop (gizzard) rate (%) = weight of crop (gizzard) ÷ body weight of chicken × 100.

#### 16S rRNA Gene Sequencing

The 16S rRNA gene of the gut microbiota was sequenced by Gene *de novo* Biotechnology Ltd (Guangzhou, China) using High-Throughput Sequencing Technology (25).

The accession number can be found below: NCBI Sequence Read Archive (SRA); PRJNA719301.

Bioinformatics Analysis: (1) Quality control and reads assembly. Raw reads were further filtered using FASTP. Paired-end clean reads were merged as raw tags using FLSAH (26). Raw tag filtering: Noisy raw tag sequences were filtered via the QIIME pipeline under specific filtering conditions (27) to obtain high-quality clean tags. Chimera checking and removal: clean tags were searched against the reference database for reference-based chimera checking using the UCHIME algorithm. All chimeric tags were removed, and effective tags were finally obtained. (2) Operational taxonomic units (OTUs) cluster. Effective tags were clustered into OTUs with ≥97% similarity using the UPARSE pipeline (28). Venn analysis was performed in R project to identify OTUs. (3) Microbial diversity was analyzed. The alpha diversity indexes were analyzed using QIIME. The comparison of microbial alpha diversity among groups was performed *via* Kruskal–Wallis using the Vegan package in the R project (29). Beta diversity was assessed. Sequence alignment was carried out using Muscle (30). Microbial beta diversity analyses among groups were performed via Kruskal–Wallis using the Vegan package in the R project. In addition, principal coordinates analysis (PCoA) of unweighted unifrac distances was calculated and plotted in the R project. (4) Bacteria biomarker features of each group were screened by Metastats (31) and LEfSe (linear discriminant analysis (LDA) effect size) software (32). Metastats showed significantly different bacteria using *P* < 0.01 or 0.05. The value of LDA of certain microbes >2 represents that the difference is significant.

#### The Concentration of SCFAs

The concentration (mmol/100 g) of SCFAs including acetate, propionate, and butyrate in the cecum chyme was measured using the internal standard method with High Performance Gas Chromatography (Trace 1300, Thermo Fisher Scientific, America) (33). Next, the contents (mmol) of acetate, propionate and butyrate in the total cecum chyme were calculated. The content of a certain SCFA = the concentration of a certain SCFA (mmol/100 g) × the weight of the total cecum chyme (g) × 100.

### Statistical Analysis

Statistical analyses of indexes were performed using one-way analysis of variance (ANOVA) via the SPSS 22.0 software. The *T*-test was used to compare indexes between the two breeds of hens. The results are expressed as the means with pooled standard error of the mean (SEM) (34). A *P*-value < 0.05 was considered significant.

## Results

For simplicity, the “Bian hens–20 weeks” are named BHT, and the “ISA Brown hens–20 weeks” are named IBHT. BHT includes the BHT-OL, BHT-OL and BHT-OF groups, and IBHT includes the IBHT-OL, IBHT-OL and IBHT-OF groups.

### Growth Performance

There were no differences among the three groups of Bian hens (BH) and ISA brown hens (IBH) in terms of growth performance, including the body weight (BW), average daily feed intake (ADFI), and average daily gain (ADG) (*P* > 0.05).

### Laying Performance

On average, hens in the BH-OL and OH groups laid their first eggs at 21 and 22 weeks, respectively, while the hens in the OF group laid their first eggs at 24 weeks old. The OL and OH groups produced 45 and 12 eggs earlier than the OF group, respectively. The laying rates in the OL and OH groups were 14.17 and 5.36%, respectively, while the OF group had yet to lay any eggs (Table 2). This suggests that adding eubiotic lignocellulose to feed can effectively advance the laying age of BH and increase economic benefits. The egg weights and laying rates among the three groups were not significantly different during the laying period (24-27 weeks), but the egg weights of the OL and OH groups were significantly higher than those of the OF group at 24 weeks (*P* < 0.05) (Table 2).

**Table 2:** Effect of adding eubiotic lignocellulose on the laying performance of BH and IBH.

**Items**	**Age (weeks)**	**OL group**	**OH group**	**OF group**	**SEM**	***P-*value**
**BH**						
Layingage (week)		21	22	24		
**Egg weight (g)**						
	21st	36.75	-	-		
	22nd	39.41	39.00	-		
	23rd	40.83	42.00	-		
	24th	41.56^A^	40.88^A^	36.95^B^	0.60	0.001
	25th	44.02	43.31	43.73	0.36	0.74
	26th	45.27	45.15	45.93	0.18	0.19
	27th	46.95	47.25	47.02	0.08	0.34
**Laying rate (%)**						
	21st		-	-		
	22nd	7.64	3.57	-		
	23rd	14.17	5.36	-		
	24th	25.60	23.47	16.67	2.51	0.35
	25th	58.33	54.08	53.33	2.22	0.63
	26th	72.62	73.98	66.67	1.87	0.27
	27th	81.25	83.33	76.67	1.63	0.24
**IBH**						
Layingage (week)		19	19	19		
**Egg weight (g)**						
	19th	43.07	44.85	44.38	0.60	0.54
	20th	50.00	49.90	50.28	0.45	0.95
	21st	54.17^ABa^	52.80^Bb^	54.75^Aa^	0.28	0.006
	22nd	55.49	55.74	55.37	0.31	0.89
	23rd	57.48	56.46	56.25	0.39	0.41
	24th	58.01	57.50	57.15	0.48	0.78
	25th	59.09	59.30	58.15	0.51	0.65
**Laying rate (%)**						
	19th	10.92^a^	4.17^b^	15.00^a^	2.22	0.017
	20th	49.26^A^	24.76^B^	59.52^A^	4.19	0.002
	21st	81.90^A^	62.08^B^	85.41^A^	3.22	0.002
	22nd	89.65^A^	81.43^B^	88.10^A^	1.94	0.008
	23rd	98.03^A^	86.67^B^	94.76^A^	1.42	0.001
	24th	99.01^A^	92.38^B^	88.57^B^	1.15	0.001
	25th	95.79^A^	90.74^B^	90.00^B^	0.82	0.003

*In the same line, values depicted with different lowercase superscript letters or uppercase superscript letters imply significant difference (P < 0.05 or P < 0.01) among groups. -, the egg has not been laid*.

In contrast with BH, all the IBH groups laid their first eggs at 19 weeks old. There was no significant difference in egg weight among the three groups within different laying periods (19-25 weeks), but egg weights in the OL and OF groups were significantly higher than that in the OH group at 21 weeks (*P* < 0.05), and the average daily laying rates in the OL and OF groups were significantly higher than that of the OH group (*P* < 0.01) (Table 2). As such, adding a high level (4%) of eubiotic lignocellulose inhibited the laying rate of IBH.

### Development of Gastrointestinal Tract

#### Comparison of Development of Gastrointestinal Tract Among Groups

There were no significant differences among the three groups of BH, except that the cecum weight of hens in the OL group was significantly higher than that of hens in the OH group (*P* < 0.05) (Table 3). However, for IBH, the weight of the crop in the OL and OH groups was significantly lower than in the OF group (*P* < 0.01). In addition, in IBH the weight and length of the small intestine and cecum were significantly lower in the OH group than in the OF group (*P* < 0.05) (Table 3). This indicates that the addition of eubiotic lignocellulose inhibited the development of the gut in IBH.

**Table 3:** Effect of adding eubiotic lignocellulose on the development of gastrointestinal tract among groups in BH and IBH.

**Items**	**BH**			**SEM**	***P*-value**	**IBH**			**SEM**	***P*-value**
	**OL group**	**OH group**	**OF group**			**OL group**	**OH group**	**OF group**		
**Weight (g)**										
Crop	5.17	5.26	4.56	1.47	0.66	5.23^Bb^	5.24^Bb^	6.70^Aa^	0.77	0.002
Gizzard	24.75	25.47	27.51	2.85	0.36	31.45	34.13	33.11	3.62	0.47
Small intestine	53.06	56.49	52.90	8.83	0.71	76.50^ab^	71.62^b^	85.33^a^	8.36	0.045
Cecum and chyle	3.89	4.11	3.45	0.58	0.22	5.28^ab^	4.66^b^	6.09^a^	1.06	0.013
Cecum	1.74^ab^	2.15^a^	1.16^b^	0.33	0.034	3.45^b^	2.74^b^	4.00^a^	0.76	0.026
Chyle	2.15	2.42	2.30	0.39	0.82	2.19	1.92	2.10	0.82	0.87
**Length (cm)**										
Small intestine	98.33	97.88	92.5	7.77	0.51	107.58^ab^	102.79^b^	116.28^a^	11.2	0.038
Cecum	11.60	11.96	11.58	0.92	0.77	14.13^ab^	12.97^b^	15.2^a^	1.56	0.033

*In the same line, values depicted with different lowercase superscript letters or uppercase superscript letters imply significant difference (P < 0.05 or P < 0.01) among groups*.

#### Comparison of Development of Gastrointestinal Tract Between BH and IBH

**Figure 1:**
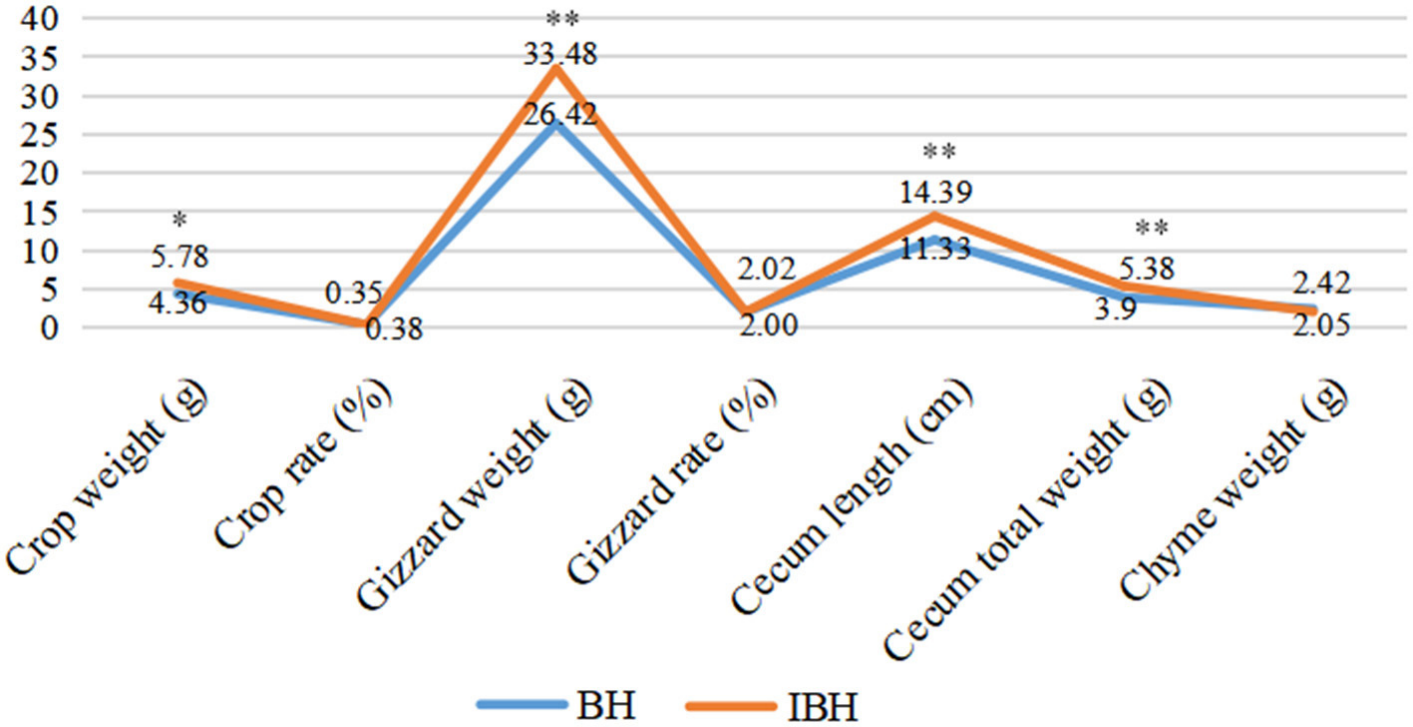
Comparison of effect of adding eubiotic lignocellulose on the development of gastrointestinal tract between BH and IBH. * or ** imply significant difference (*P* < 0.05 or *P* < 0.01).

**Figure 2:**
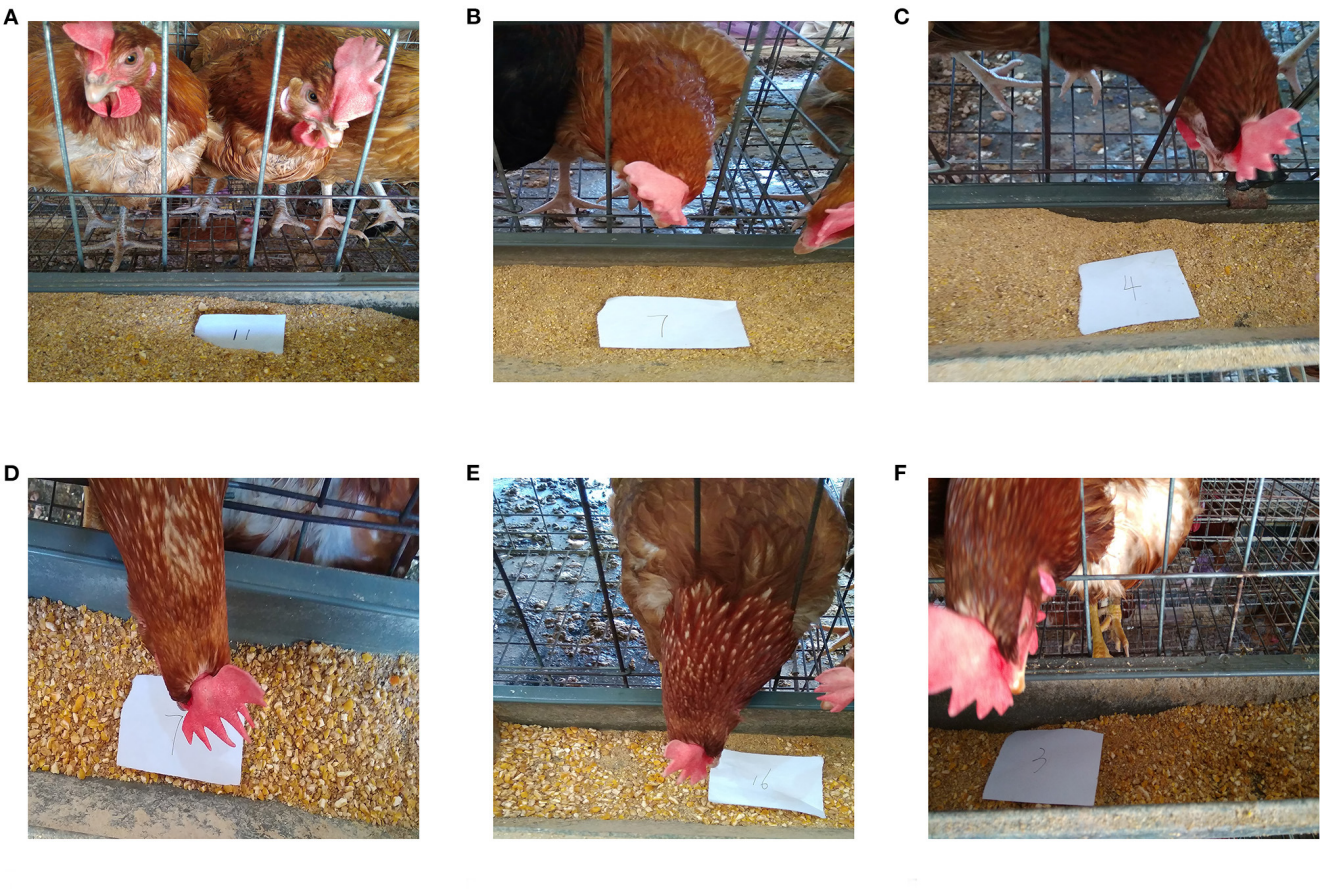
Leftovers of Bian hens–20 weeks (BHT) and ISA Brown hens–20 weeks (IBHT). **(A**-**C)** Part of the powder feed remained in the BHT's chute. **(D-F)** Part of the powder feed remained in the IBHT's chute.

The values representing the development of the gastrointestinal tract in BH were significantly lower than those of IBH (*P* < 0.05 or *P* < 0.01), except for crop ratio, gizzard ratio and cecum chyme weight (*P* > 0.05) (Figure 1). In addition, BH preferred granular corn to powder feed (Figures 2A–C), while IBH preferred powder feed and avoided granular corn (Figures 2D–F).

### OTUs and Gut Microbial Diversity

#### Comparison of OTUs and Gut Microbial Diversity Among Groups

In BH, the differences between the numbers of total OTUs and unique OTUs in the OL (1,004, 114) and OH groups (984, 102) and those in the OF group (1,007, 130) were minor. In IBH, the number of OTUs in the OH group (1,180, 208) was slightly higher than in the OL (1,057, 116) and OF groups (983, 88). There were no significant differences in gut microbial α-diversity or β-diversity among the three groups of BH and IBH (*P* > 0.05).

#### Comparison of OTUs and Gut Microbial Diversity Between BH and IBH

**Figure 3:**
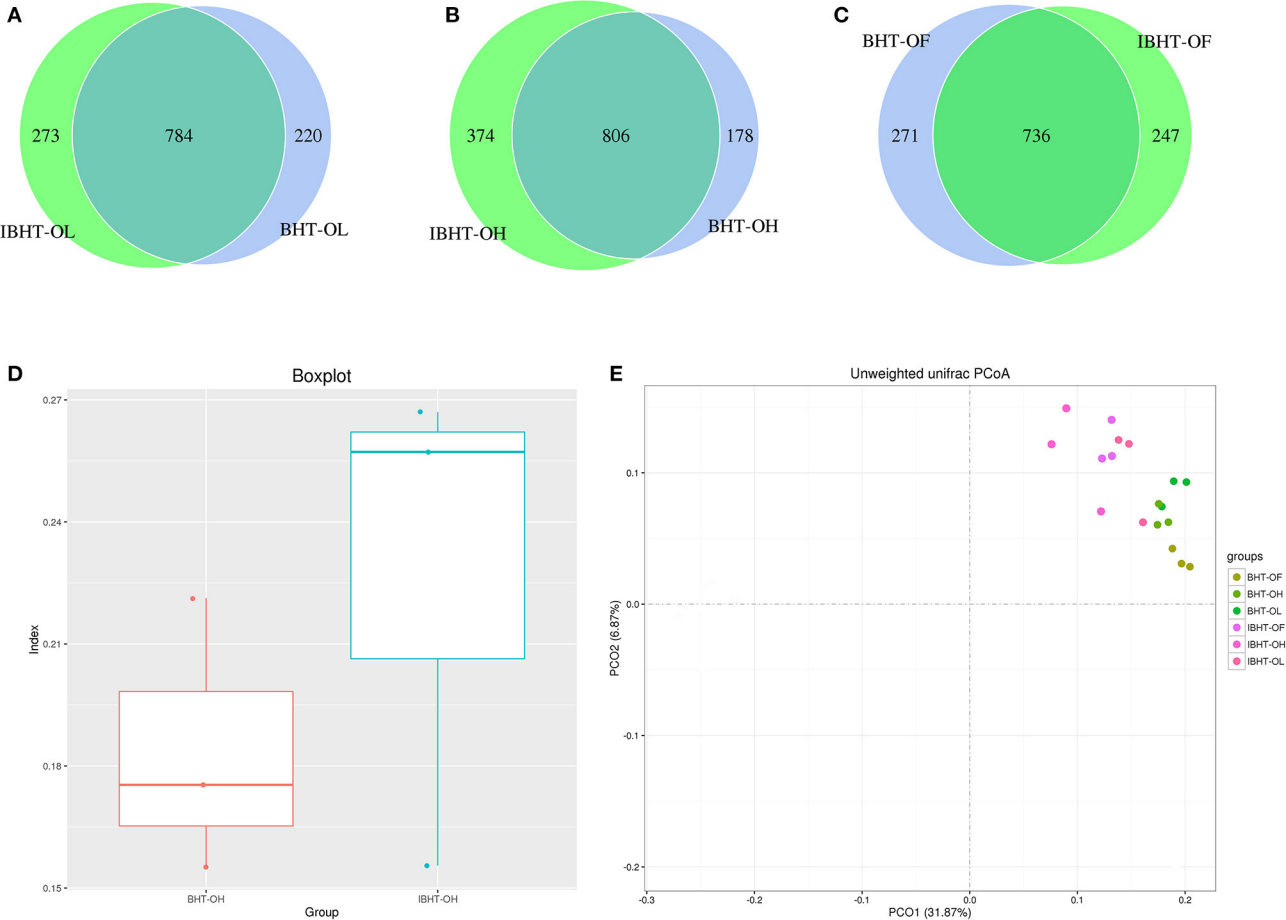
Comparison of OTUs (operational taxonomic units), PCoA (principal coordinates analysis), and gut microbial diversity between Bian Hens–20 weeks (BHT) and ISA Brown Hens–20 weeks (IBHT). **(A-C)** The Venn diagram of comparison of OTUs between BHT and IBHT. The overlapping parts show the number of OTUs shared by two groups, while the values on either side are the numbers of unique OTUs in each group, respectively. **(D)** Box plot of β-diversity between BHT and IBHT. **(E)** Principal coordinates analysis (PCoA) of samples of BHT and IBHT. Different color dots represent samples from different groups of two breeds.

The number of total and unique OTUs of BH-OH group (984, 178) were less than those in IBH-OH group (1,180, 374) (Figures 3A–C). The β-diversity of BH-OH group (0.18) was also significantly lower than those in IBH-OH group (0.23) (*P* < 0.05) (Figure 3D). The PCoA clustering graph shows that the samples were clearly clustered by breed (Figure 3E).

### Gut Microbial Composition

**Figure 4:**
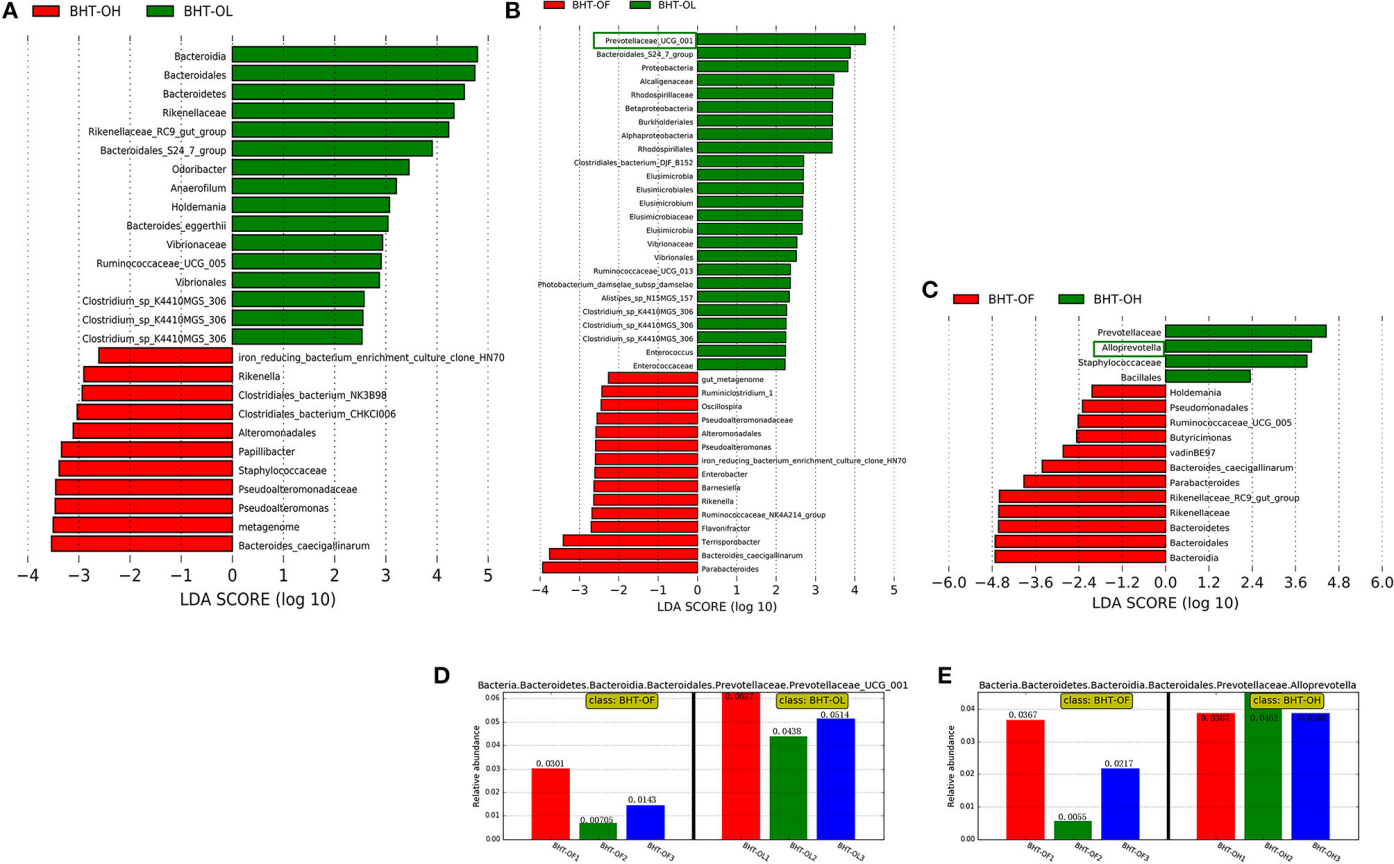
Linear discriminant analysis (LDA) among the groups of Bian hens–20 weeks (BHT) and abundance histograms of the dominant bacteria. **(A)** BHT-OL vs. BHT-OH. **(B)** BHT-OL vs. BHT-OF. **(C)** BHT-OF vs. BHT-OH. A value of LDA > 2 represents that the difference in the relative abundances of certain microbes was significant between groups. **(D)** The abundance histogram of dominant fiber-degrading genus *Prevotellaceae_UCG-001* (green frame, Figure 2B) in the OL group compared to the OF group. **(E)** The Abundance histogram of dominant fiber-degrading genus *Alloprevotella* (green frame, Figure 2C) in the OH group compared to the OF group. OL, OptiCell (OC)-low; OH, OC-high; OF, OC-free.

**Figure 5:**
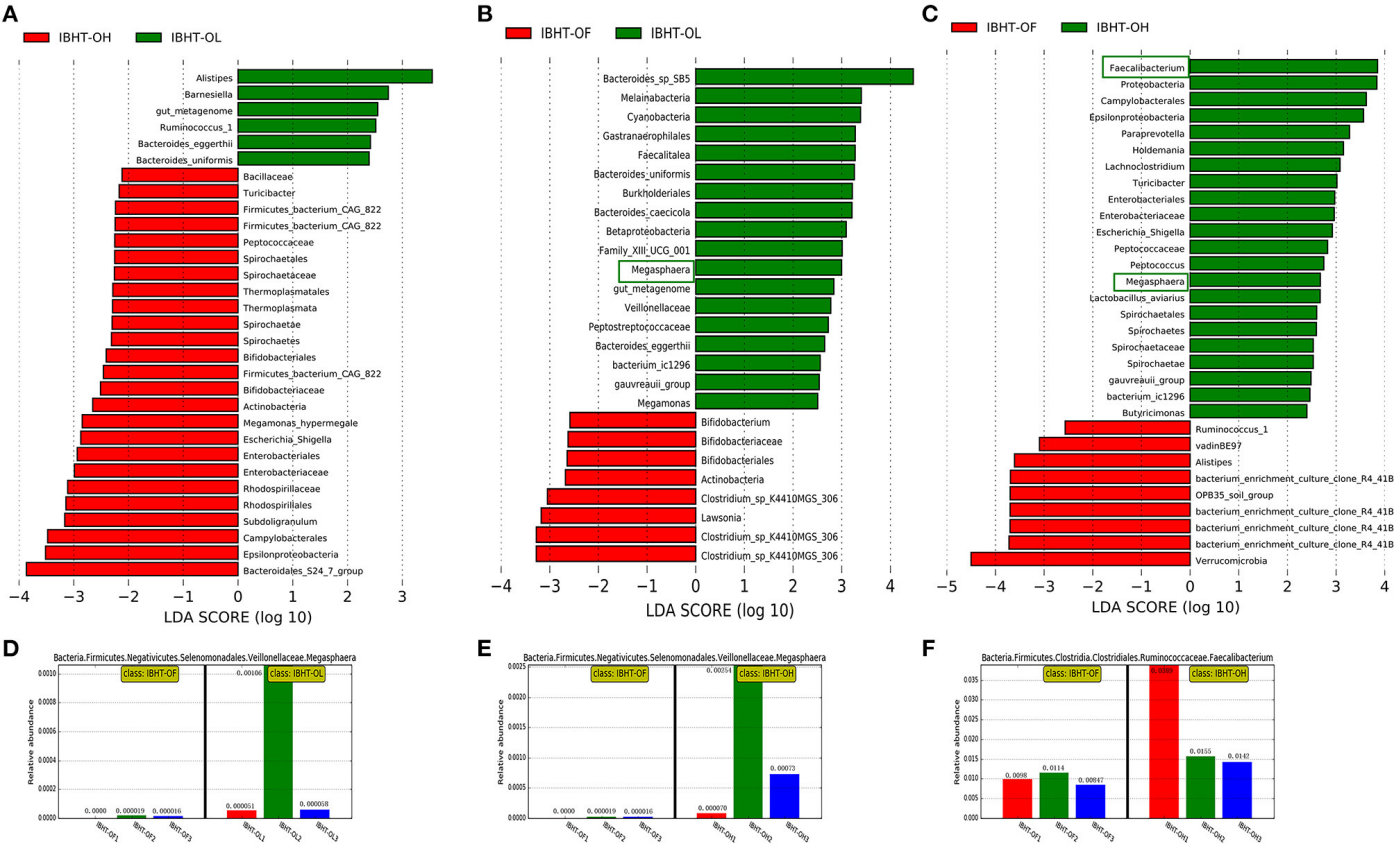
LDA (linear discriminant analysis) among groups of ISA Brown hens–20 weeks (IBHT) and abundance histograms of the dominant bacteria. **(A)** IBHT-OL vs. IBHT-OH. **(B)** IBHT-OL vs. IBHT-OF. **(C)** IBHT-OH vs. IBHT-OF. **(D,E)** The abundance histogram of dominant butyrate-producing genus *Megasphaera* in the OL group (green frame, **B**) and OH group (green frame, **C**) compared to the OF group. **(F)** The abundance histogram of dominant butyrate-producing genus *Faecalibacterium* (green frame, **C**) in the OH group compared to the OF group.

**Figure 6:**
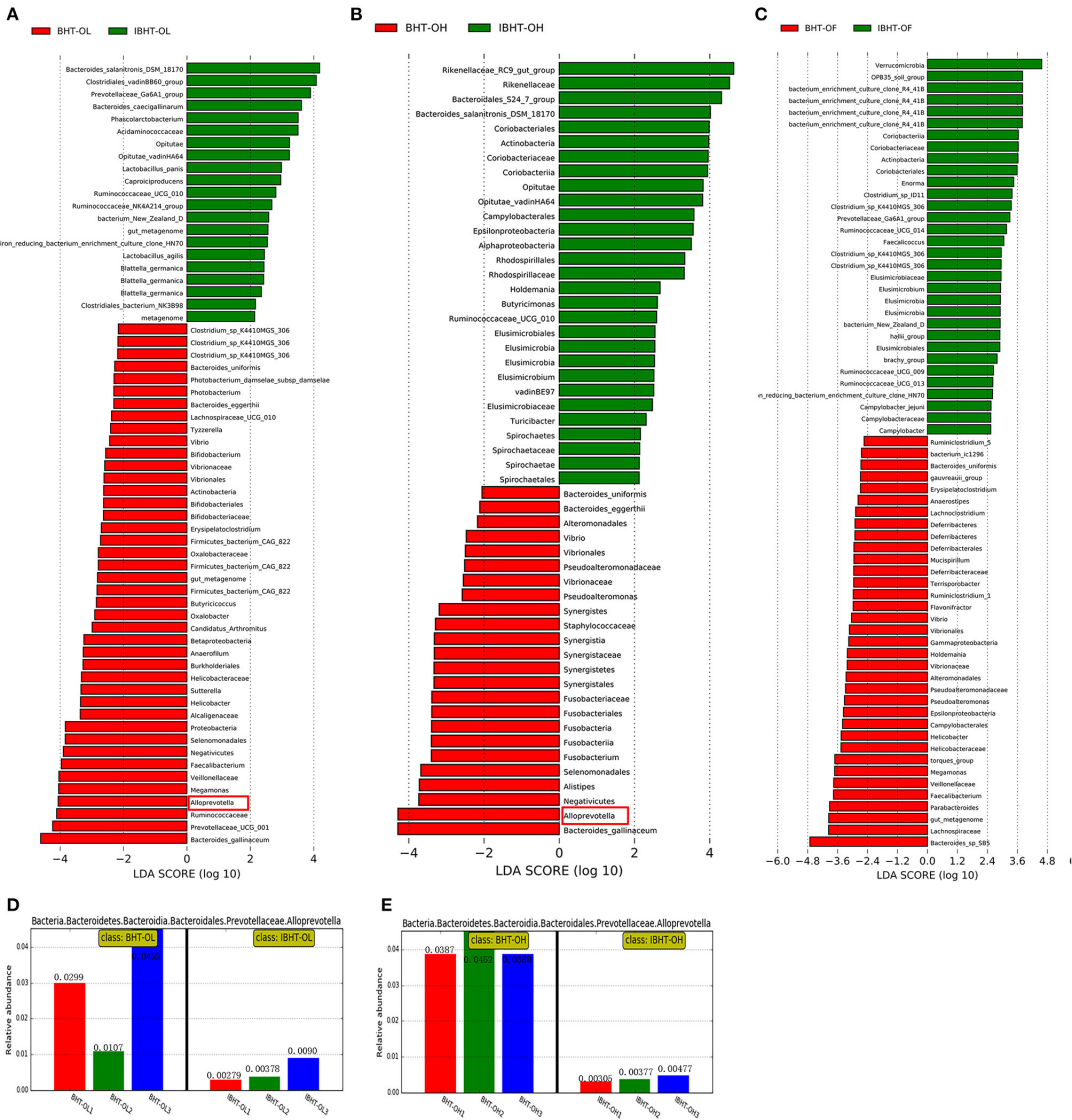
Linear discriminant analysis (LDA) between Bian hens–20 weeks (BHT) and ISA Brown hens–20 weeks (IBHT) and abundance histograms of the dominant bacteria. **(A)** BHT-OL vs. IBHT-OL. **(B)** BHT-OH vs. IBHT-OH. **(C)** BHT-OF vs. IBHT-OF. **(D,E)** Abundance histograms of dominant fiber-degradation genus *Alloprevotella* in BH compared to those in IBH.

An LEfSe of gut microbial composition was constructed using LDA (linear discriminant analysis). Figures 4–6 showed certain microbes that displayed significant differences (LDA value > 2) between groups or breeds. Fiber-degradation bacteria and SCFAs-producing bacteria with significant differences in these figures were concentrated on.

#### Comparison of Gut Microbial Composition Among the Groups

In BH, the relative abundances of the fiber-degrading genera *Prevotellaceae_UCG-001* in the OL group (5.27%) [Figure 4B (green frame), Figure 4D] and *Alloprevotella* in the OH group (4.09%) [Figure 4C (green frame), Figure 4E] were significantly greater than those in the OF group (1.72, 2.13%) (*P* < 0.05).

In IBH, the relative abundance of the butyrate producer *Megasphaera* in the OL (0.039%) [Figure 5B (green frame), Figure 5D] and OH groups (0.11%) [Figure 5C (green frame), Figure 5E] was significantly greater than that in the OF group (0.0013%) (*P* < 0.05). In addition, the relative abundance of the butyrate-producing genus *Faecalibacterium* in the OH group (2.29%) [Figure 5C (green frame), Figure 5F] was significantly greater than that in the OF group (0.99%) (*P* < 0.05).

#### Comparison of Gut Microbial Composition Between BH and IBH

The relative abundances of fiber-degrading bacterial genera *Alloprevotella* (2.86%) [Figure 6B (red frame), Figure 6D], and *Prevotellaceae_UCG-001* (5.27%) and butyrate-producing bacteria *Faecalibacterium* (2.77%) in the BH-OL group were significantly greater than those in the IBH-OL group (0.52, 1.79, 1.06%) (*P* < 0.05), respectively.

In addition, the relative abundances of fiber-degrading bacterial genera *Alloprevotella* (4.09%) [Figure 6C (red frame), Figure 6E] and butyrate-producing bacteria *Fusobacterium* (0.59%) of BH-OH group were significantly higher than those in the IBH-OH group (0.39, 0.11%) (*P* < 0.05), respectively.

Notably, *Bacteroides thetaiotaomicron*, which was one of the best fiber-degrading bacterial species, was detected in all groups of BH (0.0037, 0.0014, 0.0015%), but it was lacking in the IBH groups (0, 0, 0%). It may be unique to BH.

### Concentration of SCFAs

#### Comparison of Gut Microbial Composition Among Groups

In BH, there were no significant differences among the groups in the concentration (mmol/100 g) and content (mmol) of SCFAs in the cecum chyme (*P* > 0.05), but the levels of SCFAs in the OL and OH groups were higher than those in the OF group (Table 4). In contrast, in IBH, the level of SCFAs in the OH group was lower than those in the OL and OF groups (Table 4). In particular, the concentration of butyrate in the OH group was significantly lower than that in the OL and OF groups (*P* < 0.05). In addition, the content of SCFAs in the total cecum chyme of the OH group was also significantly lower than in the OL and OF groups (*P* < 0.01) (Table 4).

**Table 4:** Effect of eubiotic lignocellulose on the concentration of and content of SCFAs in BH and IBH.

**Groups**	**SCFAs concentration (mmol/100 g)**				**SCFAs content (mmol)**			
	**Acetate**	**Propionate**	**Butyrate**	**Total SCFAs**	**Acetate**	**Propionate**	**Butyrate**	**Total SCFAs**
**BH**								
OL group	5.00	2.59	0.77	8.36	0.100	0.052	0.015	0.17
OH group	5.17	2.37	0.90	8.44	0.112	0.051	0.019	0.18
OF group	4.14	1.44	0.57	6.15	0.092	0.032	0.013	0.14
SEM	0.31	0.17	0.35	0.58	0.0064	0.0055	0.0020	0.011
*P*-value	0.24	0.11	0.21	0.168	0.42	0.24	0.38	0.45
**IBH**								
OL group	6.42	2.22	0.74^a^	9.38	0.14^a^	0.047^a^	0.016^a^	0.20^a^
OH group	4.98	1.63	0.42^b^	7.04	0.081^b^	0.026^b^	0.0068^b^	0.11^b^
OF group	6.12	1.91	0.60^a^	8.62	0.13^a^	0.041^a^	0.013^a^	0.18^a^
SEM	0.39	0.15	0.056	0.51	0.0092	0.0037	0.0013	0.012
*P*-value	0.39	0.36	0.027	0.239	0.04	0.033	0.022	0.013

*In the same column, values depicted with different lowercase superscript letters or uppercase superscript letters imply significant difference (P < 0.05 or P < 0.01) among groups*.

#### Comparison of Gut Microbial Composition Between BHT and IBHT

The concentration (mmol/100 g) of butyrate in the IBHT-OH group was also significantly lower BHT-OH group (*P* < 0.05).

## Discussion

### Effect of Adding Eubiotic Lignocellulose on Growth Performance

In this study, the effect of adding eubiotic lignocellulose on the growth performance of Bian hens and ISA Brown hens was not significant. One reason for this result is that there were no significant differences in feed intake. This was supported by research suggesting that increasing dietary fiber has no significant effects on the ADFI of Ross 308 broilers and Hy-Line W36 layers within the 1–21d age period (6). The other reason was that eubiotic lignocellulose itself provides almost no energy, and SCFAs acetate which are produced by gut microbe degradation (11) showed no significant differences among groups in this study, resulting in no significant differences in growth performance among groups. Acetate is the main way for the body to obtain energy from dietary fiber (35). The oxidation of acetate provides 0.876 MJ/mol of energy. It can provide 1.2–10% of the total daily energy for human beings (36).

### Effect of Adding Eubiotic Lignocellulose on Laying Performance

Our results showed that the addition of eubiotic lignocellulose to the diet of BH aged 9–20 weeks significantly advanced the day of at which the first egg was laid, as well as their laying rate. Studies also have shown that the adding of 1% eubiotic lignocellulose to the diets of hens aged 30–38 weeks increases the egg production rate by 1.2% and the profit by 2% while reducing the feed conversion rate by 6% (9). However, adding 4% eubiotic lignocellulose inhibited the egg production of IBH in this study. This indicates that the tolerance of the Chinese native breed BH to eubiotic lignocellulose is stronger than that of the commercial breed IBH. One explanation may be that the addition of 4% eubiotic lignocellulose significantly inhibits the development of intestinal tissues, such as those in the small intestine of IBH, and thus reduces production performance. This is because dietary fiber is a kind of nutrient diluent, and a high fiber concentration has a negative effect on the digestion and absorption of nutrients in the intestine (37). One study also showed that 1-21-day-old laying hens had a greater capacity for fiber digestion and fermentation than broilers, which may be due to the relatively long intestinal tracts of laying hens (6).

The other reason may be that adding 4% prebiotic lignocellulose had little effect on the cecum of BH but inhibited the development of the cecum and reduced the total weight of the cecum chyme, resulting in the lower SCFA contents in the total cecum chyme. Cecum is the main contributor to fiber degradation and fermentation in chickens (38). SCFAs fermented from dietary fiber can act as energy and carbon sources for poultry (39). Chicken can derive about 8% of their energy from SCFAs (40), while ostriches can derive as much as 76% and promote the laying performance (41). SCFAs can also enhance host immunity. Therefore, the lower SCFA contents of the 4% group of IBH may lead to a poor laying performance. The mechanism enabling the BH to have a greater tolerance to high levels of eubiotic lignocellulose than IBH needs further exploration.

### Effect of Adding Eubiotic Lignocellulose on Development of the Gastrointestinal Tract

In the present study, the addition of a high level of eubiotic lignocellulose (4%) improved the development of the cecum in BH. This was supported by research which reported that consumption of a high fiber diet can also increase the relative length and weight of the chicken cecum (3). However, added 4% eubiotic lignocellulose significantly inhibited the development of the gastrointestinal tract, including the cecum, in IBH. This further supports the idea that BH have a stronger tolerance to dietary fiber than IBH. This effect may be linked to the different feeding habits of the two breeds of chicken. Poultry often pick larger food particles, leaving powder (42, 43). It was found that feeding with whole grain increased the gizzard weight of broilers. On the contrary, the gizzards of poultry fed a fine-grain diet were underdeveloped (44). Interestingly, in this experiment, BH preferred granular corn, while IBH preferred powder. This preference may have helped to stimulate the development of the crop and gizzards of BH, resulting in the absence of a significant difference in crop and gizzard rate between BH and IBH.

### Effect of Adding Eubiotic Lignocellulose on the Gut Microbiota and SCFAs

In this experiment, the addition of eubiotic lignocellulose did not increase the microbial diversity, but it did increase the relative abundances of the fiber-degrading bacteria *Prevotellaceae_UCG-001* and *Alloprevotella*, which are homologous with the *Prevotella* in BH. *Prevotella* is an excellent fiber-degrading bacterial genus with a strong fiber-degrading ability in Bacteroidetes (45). Some research has also shown that the consumption of dietary fiber can increase the abundance of *Prevotella* (46, 47). Therefore, although there were no great differences in concentrations of SCFAs among the BH groups, the levels of SCFAs in the OL and OH groups were higher than those in the OF group.

Moreover, in this study, the addition of 4% eubiotic lignocellulose increased the concentrations of the butyrate-producing bacteria *Megasphaera* and *Faecalibacterium* in the IBH group compared with the OF group. Butyrate-producing bacteria *Faecalibacterium* can ferment glucose into butyrate (22, 48). However, because of the lower cecal length and chyme weight, the butyrate concentration (mmol/100 g chyme) and SCFAs content (mmol) in the total cecum chyme of the OH group were lower than in the OF group. This may have caused the poor laying performance of this group. Currently, most research focuses on the effects of the concentration of SCFAs on the host. In fact, the effects of the SCFAs content in total chyme on the nutrition and health of the host should be emphasized. However, it seems impractical to sample and weigh the total chyme levels in the colons of humans or the rumen of cattle.

### Effect of Breeds on the Gut Microbiota and SCFAs

In this experiment, the relative abundances of the fiber-degrading bacteria genus *Alloprevotella* and the butyrate-producing bacteria *Fusobacterium* in the BH-OH group were significantly greater than in the IBH-OH group, resulting in the effective degradation and fermentation of lignocellulose into butyrate. Furthermore, the concentration of butyrate in the BH-OH group was significantly higher than that in the IBH-OH group. Butyrate is the preferred raw material for intestinal cells (49). About 95% of butyrate is absorbed into epithelial cells, which is then rapidly oxidized into ketones for ATP synthesis, providing ~70% of their energy (23) and promoting epithelial cell proliferation (50). Therefore, one reason for the poor development of the cecum in the IBH-OH group may have been that the low content of butyrate failed to provide sufficient energy for the epithelial cells.

In addition, in the present study, the excellent fiber-degrading bacterial species *Bacteroides thetaiotaomicron* was only detected in BH, but was lacking in IBH. *B. thetaiotaomicron* is one of the best fiber-degrading bacteria. More than a quarter of this genome can motivate the degradation of polysaccharides (such as rhamnogalacturonan II (RG-II), which is known to be the most complex glycan in pectin) into monosaccharides (15, 51). These differences should also be reasons that the tolerance of BH to eubiotic lignocellulose was greater than that of IBH. This was also consist with some reports which showed that the gut microbiota in different chicken breeds was usually different (8, 52, 53). That was maybe because the gut microbiota and their hosts have co-evolved for a long time, and some microbiota only colonize specific hosts. For example, Japanese people who eat lots of sushi carry the unique bacteria *Bacteroides plebeius*, which degrade Porphyra polysaccharide, while North Americans lack such bacteria (54).

## Conclusion

The tolerance of BH to eubiotic lignocellulose was shown to be greater than that of IBH. Adding 2–4% eubiotic lignocellulose is appropriate for BH, while 0–2% eubiotic lignocellulose is appropriate for IBH.

## Data Availability Statement

The datasets presented in this study can be found in online repositories. The names of the repository/repositories and accession number(s) can be found below: https://www.ncbi.nlm.nih.gov/sra/PRJNA719301.

## Ethics Statement

The animal study was reviewed and approved by this experiment was approved by the Shanxi Agricultural University Animal Experiment Ethics Committee (license number: SXAU-EAW-2017-002Chi.001).

## Author Contributions

YY: conceptualization, resources, and funding acquisition. LH: methodology and visualization. BS and LH: formal analysis, investigation, and data curation. BS: writing—original draft preparation, writing—review, and editing. YY and BS: project administration. All authors contributed to the article and approved the submitted version.

## Funding

This research was funded by the Construction of Animal Husbandry Key Project of 1331 Project in Shanxi Province (J202011315) and the Construction of Double First-Class Key Disciplines of Animal Husbandry (J202111303) and the Modern Mountain Region Ecological Poultry Innovation Talent Team in Zunyi City (Zunyi Science and Technology Talents [2021] No. 5).

## Conflict of Interest

The authors declare that the research was conducted in the absence of any commercial or financial relationships that could be construed as a potential conflict of interest.

## Publisher's Note

All claims expressed in this article are solely those of the authors and do not necessarily represent those of their affiliated organizations, or those of the publisher, the editors and the reviewers. Any product that may be evaluated in this article, or claim that may be made by its manufacturer, is not guaranteed or endorsed by the publisher.
